# Association between Knowledge of Sexually Transmitted Infections and Sources of the Previous Point of Care among Nigerians: Findings from Three National HIV and AIDS Reproductive Health Surveys

**DOI:** 10.1155/2020/6481479

**Published:** 2020-01-02

**Authors:** Imran O. Morhason-Bello, Adeniyi F. Fagbamigbe

**Affiliations:** ^1^Department of Obstetrics and Gynaecology, Faculty of Clinical Sciences, College of Medicine, University of Ibadan, Nigeria; ^2^Department of Epidemiology and Medical Statistics, Faculty of Public Health, College of Medicine, University of Ibadan, Nigeria

## Abstract

*Background. *Adequate knowledge of sexually transmitted infections (STIs) is critical for effective control of disease. Health education/counselling at the point of care provides ample opportunities to improve knowledge of patient seeking treatment. There is no study from Nigeria that investigates association between sources of previous point of care of STI and quality of knowledge of people on STI. We hypothesised that previous treatment of STI will be associated with better knowledge of STI and HIV infection. *Methods.* Three consecutives nationally representative cross-sectional surveys on HIV and AIDS Reproductive Health in Nigeria, conducted in 2005, 2007, and 2012 were analysed. Outcome measures were knowledge of STI only, and a combined knowledge of STI and HIV transmission and prevention. We designed a knowledge scale of 14-item questions for STI and 41-item questions for STI and HIV. Logistic regression was used to identify risk factors at 5% significance level. *Results. *Knowledge of STI increased from 13.4% in 2005 to 15.0% in 2007 to 26.5% in 2012. Respondents that received treatment from pharmacy and patient medicine vendors had higher odds of good knowledge of STI than those who did not receive any treatment (aOR = 2.55) in 2005. In 2012, respondents treated at health facilities were over two times likely to have good knowledge of STI and HIV transmission and prevention (aOR = 2.35). STI positive individuals in the highest economic class were two times likely to have good knowledge of STI and HIV transmission and prevention than those in the lowest class. *Conclusion. *Participants that previously sought care from health facilities, pharmacy, and patient medicine vendors had better knowledge of STIs and HIV infection prevention and transmission than those who sought care from unorthodox sources. We recommend a national awareness creation on STI prevention including provision of information on safe point of care for STIs in Nigeria.

## 1. Background

The WHO in 2018 estimated that one million people acquire sexually transmitted infections (STI) daily [1], and half a billion people fall sick from one of the four curable STIs (Chlamydia, gonorrhoea, syphilis, and trichomonas) each year [1]. Majority of curable and incurable STIs—herpes simplex virus and human papillomavirus—burdens are in low and middle-income countries, and Africa has the highest burden [2].

Literature is replete with instances to show that the risk of HIV infection acquisition multiplies 10 times in those with STIs [3, 4]. The negative health consequences of STI on several components of reproductive health include infertility, cervical cancer, chronic pelvic pain, recurrent pregnancy loss, preterm labour, and newborn/child health complications [5–7]. The economic burden of STI is also huge even in low and middle-income countries [7]. The high burden of HIV in Africa was reported to be partly due to the high rate of untreated and poorly treated STIs [8, 9]. Currently, Africa account for 20%–35% of the global burden of curable and noncurable STIs [9]. In Nigeria, there is no national figure for STI prevalence, however, previous studies reported a range of prevalence of curative STIs among the low-risk population to be 0%–18% [10, 11] and 23% among sex workers [12, 13]. The average national seroprevalence of HIV infection in among general population aged 15–49 years in Nigeria is 1.4%, with a higher prevalence among high-risk populations (sex workers, men who have sex with men, and drug users) [14, 15].

Due to the weak health infrastructure and lack of manpower, most countries in Africa have limited specialised clinics for prevention and treatment of STIs [16, 17]. Some studies in Africa showed that people with STIs patronise pharmaceutical shops, patent medicine sellers, and traditional healers [18, 19]. Additionally, adolescents and young people tend to engage in self-medication to avoid embarrassment and stigma at routine health clinics. The general control of STIs involves case management, counselling, and behavioural interventions, investing on rapid point of care diagnostic tests, anti-microbial treatment, development of vaccines, and other related interventions[20–23]. Counselling and behavioural intervention provides opportunity for caregiver and clients to interact and discuss on ways to prevent recurrence of STI and promote compliance to management protocol [24]. At the community level, behavioural change communication campaigns using cultural sensitive strategies helps to promote awareness and knowledge of STIs and potentially reduce risk within the population [24].

Knowledge acquired by people from their previous treatment of STI at health facility had been associated with positive lifestyle modification. Some studies showed that knowledge of STIs may be associated with previous treatment [25–27]. For example, a South African study reported that 36% adolescents in Eastern Cape knew about STIs from their healthcare professionals/clinic [25]. Although, there are studies that reported men and women mentioned point of care as one of the sources of information of STIs [28–30], but none of these studies compared the knowledge of these participants with their previous points of care for STI. Assessment of knowledge of STIs in people with previous experience is a useful method to assess the quality of care received. Such assessment can also provide some insight into the quality of counselling that was offered during the previous treatment. The objective of this study was to use a nationally representative data to test for association between previous point of care of STIs and knowledge acquired on STIs and HIV infection transmission and prevention as a proxy for quality of care. Our hypothesis is that previous access to STI point of care would positively improve knowledge of STI and HIV transmission and prevention.

## 2. Methods

### 2.1. Study Setting

This study used the data from three consecutive nationally representative cross-sectional surveys on HIV and AIDS Reproductive Health in Nigeria. As part of the efforts of Federal Ministry of Health (FMOH) in Nigeria to generate reliable data for an effective programme, it collaborated with other key stakeholders and conducted Nigeria's first National HIV and AIDS Reproductive Health Survey (NARHS) in 2005, 2007, and 2012. The surveys used a similar methodological approach, including data collection instrument, survey methods, analysis plan, and report writing for the surveys except that serological component was added to the 2012 survey [31]. The present study used the data from the last three surveys' information on HIV/AIDS and other reproductive health issues. Pretested questionnaires administered by trained interviewers were used.

### 2.2. Study Design

The surveys were cross-sectional and nationally representative. The surveys adopted stratified multistage cluster sampling technique to select a probability sample of women aged 15–49 years and men aged 15–64 years from households in rural and urban areas in all the 36 states and the Federal Capital Territory (FCT) Nigeria. Nigeria has 774 local government areas and an estimated population of 190.9 million in 2017. The FCT is the administrative capital of Nigeria, centrally located with population of 800,000 people and has only 5 local government areas.

### 2.3. Sampling and Sample Sizes

The Local Governments Areas (LGAs) in each state were stratified into rural and urban localities from which some urban and rural LGAs were identified. Four-stage cluster sampling was used to select eligible persons in each of the surveys. Stage 1 involved the selection of rural and urban LGAs from each state and the FCT. Stage 2 involved the selection of Enumeration Areas (EA) known as clusters within the selected LGAs. Households were then selected from the clusters at stage 3, while individuals who are the sampling units were sampled from the households at the stage 4. While 20 and 10 individuals were sampled from urban and rural EAs, respectively, in the 2005 and 2007 surveys, thirty-two individuals were sampled from each of the EAs irrespective of urban or rural areas. The overall sample size for 2005, 2007, and 2012 survey was 10255, 11822, and 35520, respectively. In all 10,081, 11,521 and 31235 participated in the 2005, 2007, and 2012 surveys respectively, 1.65%, 2.5%, and 12.1% nonresponse rates.

### 2.4. Inclusion and Exclusion Criteria

Only 601, 728, and 1861 respondents who have experienced at least one episode of STI within 12 months preceding 2005, 2007, and 2012 surveys, respectively, were included in the further analysis after the determination of STI prevalence among all individuals sampled. The individuals identified as having experienced STI in the last 12 months before the survey are those who answered in affirmative (yes) to any of the following questions: “Have you had an abnormal genital discharge during the past 12 months?”, “Have you had genital itching during the past 12 months?”, “Have you had a genital sore/ulcer during the past 12 months?” and “Have you had genital rash during the past 12 months?”. However, the last question was not asked in the 2005 and 2007 surveys.

### 2.5. Dependent Variable

The outcomes of interest in this study are the knowledge of STI only, and a combined knowledge of STI and HIV transmission and prevention. We used 14 knowledge questions to determine knowledge of STI. The details of the questions are presented in Table 1.

A total of 41 knowledge scale consisting of 14 STI knowledge scale and 27 questions on HIV transmission and prevention and PMTCT were used to assess knowledge of STI and HIV (Table 1). We assigned a score of “1” to each correct response and “0” to each wrong response. We calculated the total and mean knowledge scores and grouped the STI positive individuals into two groups according to their knowledge score: Those who scored 50% or above were regarded as having good knowledge and those with less than 50% scores as having poor knowledge as used in an India study [32].

### 2.6. Independent Variables

The exposure variable in this paper is the "previous access to STI point of care" measured by “facilities visited for treatment”. The confounders are, “sex of respondents”, categorised as male and female; “educational attainment”, categorised as No Formal, Primary/Qur'anic, Secondary and Higher education, “economic status”, this was categorized into poorer, middle, and richer wealth quintiles, the economic status was not available in 2005 and 2007 as relevant information to compute the variable was not collected; “location of residence” as rural and urban, “geopolitical zones” categorized into: North Central, North East, North West, South East, South South, and South West; “marital status” categorized as Currently married, Widowed, and Never Married, “religion” categorized into Islam, Other Christian, Catholic, and Others. We grouped the type of facilities where care was sought into three: (1) Health facility (Government, Workplace, and private health facility), (2) Pharmacy/Patent Medicine vendors, and (3) Others (Faith-based and Traditional healers), while those that did not seek care in any facility were grouped as “none”. All the variables considered in this study are categorical.

### 2.7. Statistical Analyses

The data analysis included descriptive analysis with frequency distribution for knowledge scores. Bivariate analyses were carried out using Pearson Chi-square (*x^2^*) test of association. We used logistic regression to model relationship between the outcome variables and independent variables. We controlled for variables which have a significant bivariate relationship with the outcome variables in multiple logistic regression. This was done to ensure that the effects of all other variable predictors were taken into account while estimating the effect of each explanatory variable. We checked for multicollinearity among variables and removed the highly correlated variables using partial correlation.

The data were weighted to reflect differences in population sizes of each state in Nigeria. The intra-cluster correlation was minimized by the use of effective sample size and complex survey data analysis mechanism in STATA 15. Statistical significance was set at 5%.

## 3. Results

Of the 10255, 11822, and, 35520 participants in 2005, 2007, and 2012 surveys, respectively, 54% were males in 2005 and 2007 compared with 50% in 2012. The rural dwellers among the participants were 64% in 2005, 65% in 2007, and 68% in 2012. In 2005, 38% had secondary education compared with 40% in 2007 and 39% in 2012. There are 601, 728, and 1861 respondents who have experienced at least one episode of STI within 12 months preceding 2005, 2007, and 2012 surveys, respectively. The prevalence of STI in Nigeria seemed stable at 6.0%, from 6.0% in 2005 to 6.3% in 2007 and back to 6.0% in 2012. The prevalence was consistently higher among respondents aged 20–24 years at 8.0%, 9.2%, and 7.8% across the survey years. Female respondents also had higher prevalence than the males at 8.9% vs 3.4%, 9.8% vs 3.3%, and 7.7% vs 4.3% in 2005, 2007, and 2012, respectively (Table 2).

We present the distribution STI positive respondents and the bivariate association between Frequency of genital discharges, knowledge of STI, and HIV/AIDS by facility attended and STI types in Table 3. Of the 601 STI positive respondents within the 12 months preceding the 2005 survey, 40.9% did not visit any health facility for treatment, 30.8% visited health facilities, 16.0% visited the pharmacy and patient medicine vendors, while 12.3% visited the traditional birth attendants. About 44.8% and 41.5% did not visit any facility within the twelve months preceding the 2007 and 2012 surveys, respectively. Of the 14-point scale score for knowledge of STI transmission and prevention, the mean score (mean ± Semi-Interquartile Range) was 3.70, ± 2.5, 2.59 ± 2.5, and 3.29 ± 3.0 in 2005, 2007, and 2012 surveys, respectively, and 21.5 ± 3.5, 18.6 ± 3.5 and 19.0 ± 5.5 out of 41 points for overall knowledge within the same period. The percentage of having good knowledge of STI rose from 13.4% in 2005 to 15.0% to 2007 and further to 26.5% in 2012 but the percentage overall knowledge of STI and HIV was about 62% throughout the period considered in this study. Individuals who had only one episode of any of genital discharge, itching, or sore had good knowledge of STI than those who had more episodes as shown in Table 3.

Also, Table 4 shows the distribution of respondents with STI and the bivariate association between respondents' characteristics and knowledge of STI and HIV/AIDS. We found the knowledge of STI to increase significantly with increasing age of the respondents, 9.6% for 15–19 years old to 23.1% among those aged 50–64. Zone of residence of respondents was significantly associated to their knowledge of STI as well as knowledge of STI and HIV transmission and prevention with highest proportion of good knowledge in South East (33.3%, 20.4%, and 31.5%) and least in North West (13.8%, 12.2%, and 25.4%) in 2005, 2007, and 2012, respectively. Economic status of the respondents also influenced their knowledge of STI significantly in 2012 with 31.3% among those in the higher class and 21.8% in the lowest class. Similarly, respondents in the lowest class had 47.2% good knowledge of STI and HIV transmission and prevention compared with 75.4% among those in the highest class. Educational attainment of the respondents and religious inclination were significant to the level of knowledge of STI and HIV transmission and prevention.

Table 5 shows the outcome of the bivariate logistic regression models. In 2005 STI positive respondents who visited facilities were two times likely to have good knowledge of STI than those who did not OR = 2.07, 95% CI: 1.31–3.29. The odds were similar in 2007 and 2012 (Table 5). However, those that got treatments from pharmacists and patient medicine vendors had thrice higher odds of having good knowledge of STI than those that did not go for treatment. The odds of having good knowledge of STI and HIV transmission and prevention increased between 2005 at 2.23, 2007 at 2.13, and then 2.56 in 2012 than those who did not get any treatment. The odds of having good knowledge of STI among STI positive respondents aged 50–64 compared to those aged 15–19 years reduced from 2.84 in 2005 to 1.54 in 2012. On having good knowledge of STI and HIV transmission and prevention, the odds fell from 2.93 in 2005 to 1.57 in 2012 among those aged 50–64 compared to those aged 15–19 years. Also, higher odds of good knowledge of STI and STI and HIV transmission and prevention were found among respondents in the highest economic class, married respondents, Catholics, and other Christians as shown in Table 5.

Table 6 shows the outcome of the multiple logistic regression of determinants of good knowledge of STI. While adjusting for other factors, STI positive respondents who got treatment from Pharmacy and patient medicine vendors had higher odds of good knowledge of STI than those who did not get any treatment (aOR = 2.55, 95% CI: 1.32–4.91) in 2005. In 2012, respondents who got treatment from health facilities were over twice likely to have good knowledge of STI and HIV transmission and prevention (aOR = 2.35, 95% CI: 1.83–3.01). STI positive individuals that were from the highest economic class were double likely to have good knowledge of STI and HIV transmission and prevention than those in the lowest class.

## 4. Discussion

The study highlights that previous point of care of STI is associated with knowledge of STIs and HIV infections among men and women of reproductive age group in Nigeria. Specifically, participants that received care from the health facility, pharmacy, or patient medicine vendor tend to have good knowledge of STIs and HIV infections compared to those that did not receive any form of treatment in the three national surveys. Good knowledge of STIs was associated with being a male participant in the 2007 survey report. During the 2005 survey, participants that were 25–49 years old, from the Northeast region of the country and with higher educational status above primary school had good knowledge of STIs. However, findings from 2012 survey showed that good knowledge of STIs increased with the age of participants and among those that were Christians. The proportion of participants with good knowledge of STIs including HIV infections was high among those aged 25–49 years in the 2005 and 2007 surveys. However, findings from the 2012 survey showed increasing odds of good knowledge of both STIs and HIV infections with age, education, and economic status of participants.

The knowledge of STIs including HIV among people in the community is crucial because it provides baseline information, help to profile and identify potential knowledge gap, and assist policy makers and programme planners to prioritise interventions to reduce the burden of the infection [1]. The point of care of STIs and HIV infections remain a strategic avenue to provide information that could improve knowledge and possibly promote preventive habits [1, 33]. Similarly, in this study, we found that previous visit to a health facility or pharmacy shops or patent medicine vendor was associated with a higher proportion of participants with good knowledge of STIs and HIV infections. There were increasing reports of patronage of pharmaceutical shops and patient medicine vendors as a fashionable alternative to healthcare facilities in Nigeria [30, 34]. The plausible reason for this changing attitude might be due to the general perception of better treatment of clients including confidentiality at these outlets as well as unrestricted access and less waiting time. However, a study that will specifically investigate reasons behind people's preferred choice of seeking STI care at pharmacy and patent medicine stores in Nigeria is needed.

We found a worrisome trend in the three surveys that a large number of participants that had STIs did not visit any treatment facilities in the previous 12 months. Furthermore, men and women were seeking care for STIs at faith-based homes and traditional birth attendants. It is not clear whether the faith-based homes had healthcare workers as medical staff. Seeking healthcare from alternative traditional or faith-based sources for different diseases or infections is very common in Africa [29, 35–37]. Studies have shown that attitudes of people toward healthcare might be influenced by belief or religious reasons [35–37]. Perhaps some of these alternative centers might be useful to disseminate information on prevention of diseases including STIs and HIV rather than allowing them to offer definitive treatment.

The findings of this study should be interpreted with caution because of the following limitations: We defined previous point of care of STI based on the question that was asked whether participant visited a facility in the previous 12 months for their STI symptoms. This question has a potential risk of recall bias and there may also be social desirability bias from participants. We assumed that counselling or health education should be part of treatment protocol. There was no question in the data set that clarifies whether counselling took place or not. Our assessment of STI symptoms was based on response of participants to questions asked by interviewers. Notwithstanding, the study provides a national information on knowledge of STIs including HIV infection over a ten-year period. We used multiple questions to assess knowledge score that covered a wide range of information on STI and HIV infection risk, prevention, and transmission [5–7, 38].

## 5. Conclusion

The study showed that men and women with STI symptoms visit either the healthcare facility or pharmacy or patient medicine vendor outlets, and these respondents tend to have good knowledge of STI and HIV infection risk, prevention and transmission. A significant proportion of participants still seek care for STIs from unorthodox treatment facilities in Nigeria. We recommend mass mobilisation and awareness creation on prevention of STIs in the community and provide information on safe point of care for STIs in Nigeria.

## Figures and Tables

**Table 1:** List of questions used to compute the knowledge of STI and HIV transmission and prevention.

SN	Issue	2005	2007	2012
A	*STI Knowledge*			
1	Can STIs prevent a woman from getting pregnant in future?	√	√	√
2	Can STIs prevent a man from fathering children in future?	√	√	√
3	Symptoms of STIs in women? Lower abdominal pain	√	√	√
4	Symptoms of STIs in women? Genital discharge	√	√	√
5	Symptoms of STIs in women? Foul smelling discharge	√	√	√
6	Symptoms of STIs in women? Burning pain on urination	√	√	√
7	Symptoms of STIs in women? Genital ulcers/sores	√	√	√
8	Symptoms of STIs in women? Swellings in groin area	√	√	√
9	Symptoms of STIs in women? Itching	√	√	√
10	Symptoms of STIs in women? Painful Sexual Intercourse	√	√	√
11	Symptoms of STIs in men? Genital discharge	√	√	√
12	Symptoms of STIs in men? Burning pain on urination	√	√	√
13	Symptoms of STIs in men? Genital ulcers/sores	√	√	√
14	Symptoms of STIs in men? Swellings in groin area	√	√	√
	HIV/AIDS & STI Transmission and Prevention			

B	*How can a person get the virus that causes AIDS?*			
1	Sexual Intercourse	√	√	√
2	Blood transfusion	√	√	√
3	Mother to unborn child	√	√	√
4	Sharing toilets	√	√	√
5	Sharing sharp objects like razors	√	√	√
6	Sharing needles	√	√	√
7	Sharing eating utensils	√	√	√
8	Mosquito bites/bed bugs	√	√	√
9	Witchcraft	√	√	√
10	Kissing	√	√	√
11	Hugging	√	√	√

C	*What can a person do to avoid getting the virus that causes AIDS?*			
12	Staying with one uninfected partner	√	√	√
13	Using condoms every time	√	√	√
14	Abstaining from sex	√	√	√
15	Delaying the onset of sexual intercourse	√	√	√
16	Avoiding sex with CSWs	√	√	√
17	Reducing number of sexual partners	√	√	√
18	Avoiding sex with people who have many sexual partners	√	√	√
19	Avoid sharing of sharp objects like needles, razors	√	√	√
20	Praying to God	√	√	√
21	Using antibiotics	√	√	√
22	Seek protection from a traditional healer	√	√	√

D	*Can the virus that causes AIDS be transmitted from mother to child?*			
1	During pregnancy	√	√	√
2	During delivery	√	√	√
3	By breastfeeding	√	√	√
4	Used any drug to prevent MTCT			√
5	Aware of ART			√

**Table 2:** Distribution of respondents and the Prevalence of STI by respondents characteristics.

	Distribution of all respondents			Prevalence of STI		
Variable	2005	2007	2012	2005	2007	2012
	*N* = 10081	*N* = 11521	*N* = 31235	%	%	%
*Age*						
15–19	24.2	21.4	16.9	^∗^4.7	^∗^5.6	4.9
20–24	18.6	18.7	15.5	8.0	9.2	7.8
25–34	26.3	28.3	29.7	7.4	7.2	7.4
35–49	23.3	24.2	29.4	5.4	5.2	5.0
50–64	7.7	7.2	8.5	1.7	1.5	3.0
*Sex*						
Male	53.7	53.6	49.5	^∗^3.4	^∗^3.3	^∗^4.3
Female	46.5	46.4	49.8	8.9	9.8	7.7
*Residence*						
Urban	34.9	34.5	31.3	^∗^7.5	^∗^7.7	^∗^5.5
Rural	64.3	65.4	68.3	5.2	5.6	6.2
*Zone*						
North Central	17.2	17.8	19.2	^∗^5.4	^∗^9.9	^∗^8.3
North East	15.0	13.3	15.8	4.7	5.0	5.7
North West	21.4	24.8	19.8	6.7	6.6	5.8
South East	12.6	11.3	13.6	3.8	4.3	5.2
South South	14.9	15.3	15.7	6.2	6.7	7.1
South West	18.9	17.3	16.0	7.9	4.3	3.1
*Economic status*						
Poorer	na	na	43.7	na	na	^∗^6.2
Middle	na	na	20.5	na	na	6.3
Richer	na	na	35.5	na	na	5.5
*Education*						
No formal education	20.4	21.4	24.3	^∗^5.3	^∗^5.1	^∗^4.9
Primary/Qur'anic	32.0	28.3	23.9	5.6	6.1	5.8
Secondary	37.8	39.5	39.2	6.2	6.8	6.8
Higher	9.9	10.9	12.3	7.5	7.5	5.8
*Religion*							
Islam	46.7	50.4	43.3	5.6	^∗^5.8	^∗^4.9
Other Cristian	37.2	36.0	41.6	6.5	6.6	6.6
Catholic	14.0	12.7	13.4	5.8	7.6	7.5
Other	1.8	1.2	1.8	6.1	4.3	5.0
*Marital Status*						
Currently married	55.2	57.0	63.8	^∗^6.5	6.4	5.9
Widowed	4.1	4.0	4.5	4.6	5.7	6.6
Never married	40.4	38.8	31.1	5.4	6.3	6.1
Average prevalence of STIs	100.0	100.0	100.0	6.0	6.3	6.0

^∗^Significant at the 5% level.

**Table 3:** Distribution STI positive respondents and association between Frequency of genital discharges, knowledge of STI and HIV/AIDS by facility attended and STI types.

Variable	Distribution of STI positive respondents			Good knowledge of STI^a^			Good knowledge of ALL^b^		
	*2005*	*2007*	*2012*	*2005*	*2007*	*2012*	*2005*	*2007*	*2012*
N	601	728	1861	601	728	1861	601	728	1861
Mean score (±SIQ)				3.70 ± 2.5	2.59 ± 2.5	3.29 ± 3.0	21.5 ± 3.5	18.6 ± 3.5	19.0 ± 5.5
*Facilities visited*									
None	40,9	44,8	41,5	^∗^11.8	^∗^10.4	^∗^18.1	^∗^61.4	^∗^54.0	^∗^48.1
Health Facility	30.8	33.5	31.2	21.1	20.1	32.9	85.4	71.3	72.6
Pharm/PMV	16.0	14.3	18.3	29.2	16.4	32.9	83.3	75.0	68.2
Others	12.3	7.4	9.0	13.5	16.7	30.4	52.7	64.8	66.7
*Had treatment*									
None	40.9	44.8	41.5	^∗^11.8	^∗^10.4	^∗^18.1	^∗^61.4	^∗^54.0	^∗^48.1
Yes	59.1	55.2	58.5	21.7	18.7	32.5	78.0	71.4	70.3
*Genital Discharge*									
Once	na	19.0	21.8	na	21.9	46.3	na	69.9	75.1
Twice	na	18.8	17.4	na	18.1	37.9	na	80.6	77.0
Thrice+	na	62.2	60.8	na	12.6	23.3	na	61.5	58.5
*Itching Frequency*									
Once	na	22.9	23.6	na	^∗^23.2	^∗^30.9	na	^∗^79.6	71.8
Twice	na	23.1	21.6	na	13.8	31.6	na	63.3	62.4
Thrice+	na	54.0	54.8	na	18.0	22.9	na	61.6	58.0
*Sore Frequency*									
Once	na	35.6	27.8	na	21.9	^∗^45.7	na	^∗^59.4	72.3
Twice	na	20.0	16.6	na	27.8	35.7	na	94.4	73.2
Thrice+	na	44.4	55.6	na	27.5	24.5	na	70.0	51.6

^∗^Significant at 5% x^2^; ^a^Knowledge of STI; ^b^Knowledge of STI, HIV & PMTCT transmission and prevention; na, Not Available; SIQ, Semi Interquartile.

**Table 4:** Distribution of respondents with STI and bivariate association between respondents characteristics and knowledge of STI and HIV/AIDS.

Variable	Distribution of Respondent with STI			Good knowledge of STI^a^			Good knowledge of ALL^b^		
	601	728	1861	601	728	1861	601	728	1861
*Age*									
15–19	19,1	19.0	13.9	^∗^9.6	13.8	^∗^22.1	^∗^65.2	55.1	55.0
20–24	25.0	27.2	20.3	16.0	13.6	19.8	71.3	64.1	62.7
25–34	32.6	32.3	36.9	20.9	13.6	28.5	78.1	57.7	60.8
35–49	21.1	19.9	24.7	21.3	20.0	30.9	64.6	64.8	62.8
50–64	2.2	1.7	4.3	23.1	16.7	30.4	84.6	58.3	65.8
*Sex*									
Male	30.6	28.0	35.7	17.4	^∗^19.6	25.4	72.3	63.2	61.5
Female	69.4	72.0	64.3	17.8	13.2	27.2	70.7	63.7	60.9
*Residence*									
Urban	43.9	42.0	28.9	19.3	^∗^10.5	29.4	^∗^78.8	^∗^69.0	^∗^71.0
Rural	56.1	58.0	71.1	16.3	18.3	25.4	65.3	59.7	57.1
*Zone*									
North Central	15.6	27.9	26.7	^∗^23.4	^∗^14.3	^∗^20.7	^∗^83.0	^∗^66.0	^∗^54.9
North East	11.8	10.5	15.1	26.8	10.5	17.5	60.6	60.5	54.3
North West	24.1	25.9	19.3	13.8	12.2	25.4	57.2	46.8	53.5
South East	8.0	7.7	11.9	33.3	20.4	31.5	79.2	85.7	69.8
South South	15.5	16.2	18.7	15.1	18.6	40.8	75.3	76.3	76.7
South West	25.0	11.8	8.3	10.0	11.6	25.2	77.3	66.3	63.2
*Economic status*									
Lowest	na	na	45.5	na	na	^∗^21.8	na	na	^∗^47.2
Middle	na	na	21.7	na	na	29.2	na	na	68.3
Highest	na	na	32.8	na	na	31.3	na	na	75.4
*Education*									
No formal education	18.1	17.3	20.0	10.1	10.3	^∗^20.0	^∗^51.4	^∗^44.4	^∗^38.0
Primary/Qur'anic	30.1	27.3	23.3	18.2	17.1	26.0	62.4	57.3	54.6
Secondary	39.3	42.5	44.7	17.4	13.9	28.1	80.9	59.3	68.8
Higher	12.5	12.9	12.0	28.0	20.2	32.3	90.7	84.0	83.4
*Religion*									
Islam	43.9	46.3	35.6	17.8	^∗^11.3	^∗^20.4	^∗^62.1	^∗^52.5	^∗^48.3
Other Christians	40.6	37.6	46.1	17.2	17.2	30.9	78.3	73.4	69.1
Catholic	13.6	15.3	16.9	19.5	21.6	28.3	81.7	73.0	67.9
Other	1.8	0.8	1.5	9.1	—	18.5	54.6	66.7	40.7
*Marital Status*									
Currently married	60.2	57.7	63.2	18.0	15.2	28.0	69.1	^∗^59.3	^∗^58.2
Widowed	3.2	3.6	5.0	15.8	23.1	25.8	68.4	57.7	60.2
Never Married	36.6	38.7	31.8	17.3	13.8	23.7	75.0	70.6	66.8
Total				13.4	15.0	26.5	62.4	63.6	61.1

^∗^Significant at 5% x^2^; ^a^Knowledge of STI; ^b^Knowledge of STI, HIV & PMTCT transmission and prevention; na, not available.

**Table 5:** Bivariate logistic regression of factors affecting knowledge of STI and other related knowledge.

Variable	Good knowledge of STI^a^ OR (95% CI)			Good knowledge of ALL^b^ OR (95% CI)		
	>2005	2007	2012	2005	2007	2012
*Facilities visited*						
None	Referent					
Health Facility	2.00 (1.18–3.38)	2.16 (1.34–3.47)	2.21 (1.72–2.84)	3.68 (2.27–5.96)	2.12 (1.49–3.01)	2.87 (2.28–3.61)
Pharm/PMV	3.08 (1.71–5.54)	1.68 (0.89–3.15)	2.22 (1.66–2.97)	3.15 (1.74–5.70)	2.56 (1.56–4.19)	2.32 (1.78–3.04)
Others	1.17 (0.54–2.53)	1.72 (0.77–3.82)	1.97 (1.35–2.87)	0.70 (0.42–1.18)	1.57 (0.86–2.86)	2.16 (1.52–3.07)
*Had treatment*						
No	Referent					
Yes	2.07 (1.31–3.29)	1.97 (1.28–3.04)	2.17 (1.74–2.72)	2.23 (1.56–3.20)	2.13 (1.56–2.89)	2.56 (2.12–3.11)
*Sex*						
Female	Referent					
Male	0.98 (0.62–1.54)	1.61 (1.05–2.47)	0.91 (0.74–1.13)	1.08 (0.73–1.59)	0.98 (0.70–1.37)	1.03 (0.85–1.25)
*Residence*						
Rural	Referent					
Urban	1.23 (0.81–1.87)	0.52 (0.34–0.81)	1.22 (0.98–1.53)	1.98 (1.36–2.86)	1.50 (1.10–2.04)	1.84 (1.48–2.29)
*Age*						
15–19	Referent					
20–24	1.80 (0.84–3.85)	0.99 (0.53–1.86)	0.87 (0.59–1.29)	1.33 (0.79–2.24)	1.46 (0.94–2.27)	1.37 (1.01–1.89)
25–34	2.50 (1.23–5.09)	0.99 (0.54–1.82)	1.41 (1.01–1.97)	1.90 (1.14–3.17)	1.71 (1.11–2.63)	1.27 (0.95–1.70)
35–49	2.55 (1.2–5.42)	1.57 (0.83–2.95)	1.58 (1.11–2.25)	0.97 (0.57–1.65)	1.50 (0.93–2.43)	1.38 (1.01–1.88)
50–64	2.84 (0.8–11.8)	1.25 (0.25–6.16)	1.54 (0.88–2.7)	2.93 (0.62–13.9)	1.14 (0.35–3.78)	1.57 (0.93–2.66)
*Zone*						
North West	Referent					
North Central	1.91 (0.98–3.74)	1.20 (0.6–2.15)	0.77 (0.5–1.06)	3.64 (1.9–6.84)	2.21 (1.4–3.32)	1.06 (0.8–1.39)
North East	2.28 (1.13–4.63)	0.84 (0.3–1.98)	0.62 (0.4–0.92)	1.15 (0.6–2.05)	1.74 (1.0–3.00)	1.03 (0.7–1.41)
South East	3.13 (1.4–6.71)	3.13 (1.5–6.41)	1.36 (0.9–1.96)	2.84 (1.3–6.13)	6.82 (3.0–15.2)	2.01 (1.4–2.87)
South South	1.11 (0.5–2.32)	1.64 (0.8–3.11)	2.03 (1.4–2.80)	2.27 (1.2–4.04)	3.65 (2.1–6.09)	2.87 (2.0–3.96)
South West	0.69 (0.3–1.42)	0.94 (0.4–2.08)	0.99 (0.6–1.53)	2.55 (1.5–4.22)	2.23 (1.3–3.80)	1.50 (1.0–2.20)
*Economic status*						
Lowest	Referent					
Middle	na	na	1.48 (1.13–1.94)	na	na	2.41 (1.88–3.09)
Highest	na	na	1.64 (1.29–2.07)	na	na	3.43 (2.73–4.31)
*Education*						
No formal education	Referent					
Primary/Qur'anic	1.99 (0.96–4.12)	1.79 (0.91–3.54)	1.41 (1.01–1.97)	1.57 (0.97–2.54)	1.68 (1.07–2.63)	1.96 (1.48–2.60)
Secondary	1.87 (0.92–3.80)	1.41 (0.73–2.71)	1.57 (1.17–2.11)	4.02 (2.44–6.60)	2.82 (1.84–4.31)	3.59 (2.78–4.63)
Higher	3.46 (1.55–7.72)	2.20 (1.03–4.72)	1.91 (1.31–2.80)	9.19 (3.88–21.8)	6.58 (3.42–12.7)	8.2 (5.44–12.36)
*Religion*						
Islam	Referent					
Other Christian	0.96 (0.61–1.52)	1.63 (1.03–2.58)	1.75 (1.38–2.22)	2.2 (1.48–3.26)	2.49 (1.77–3.51)	2.39 (1.93–2.95)
Catholics	1.12 (0.60–2.10)	2.17 (1.23–3.82)	1.54 (1.13–2.10)	2.72 (1.48–5.03)	2.44 (1.52–3.91)	2.26 (1.71–3.00)
Others	0.46 (0.06–3.69)	na	0.89 (0.33-2.39)	0.73 (0.22–2.46)	1.81 (0.33–10.0)	0.73 (0.34–1.61)
*Marital status*						
Currently married	Referent					
Widowed	0.86 (0.24–3.03)	1.67 (0.65–4.32)	0.9 (0.55–1.45)	0.97 (0.36–2.62)	0.94 (0.42–2.09)	1.09 (0.71–1.67)
Never married	0.95 (0.61–1.48)	0.89 (0.58–1.37)	0.8 (0.64–1.01)	1.34 (0.92–1.96)	1.65 (1.19–2.27)	1.45 (1.18–1.78)

^∗^Significant at 5%; ^a^Knowledge of STI; ^b^Knowledge of STI, HIV transmission and prevention and PMTCT; na, Not Available; Or, Odds ratio; CI, Confidence Interval.

**Table 6:** Multiple logistic regression of factors affecting knowledge of STI and other related knowledge.

Variables	Good knowledge of STI^a^ aOR (95% CI)			Good knowledge of ALL^b^ aOR (95% CI)		
	2005	2007	2012	2005	2007	2012
*Facilities visited*						
None	Referent					
Health facility	1.61 (0.91–2.86)	1.86 (1.12–3.07)	1.99 (1.53–2.59)	2.64 (1.56–4.47)	1.53 (1.04–2.27)	2.35 (1.83–3.01)
Pharm/PMV	2.55 (1.32–4.91)	1.50 (0.77–2.94)	1.83 (1.34–2.48)	2.71 (1.41–5.21)	2.04 (1.19–3.50)	1.93 (1.44–2.59)
Others	1.10 (0.49–2.47)	1.78 (0.78–4.07)	1.88 (1.27–2.79)	0.65 (0.37–1.17)	1.60 (0.85–3.01)	2.44 (1.66–3.57)
*Had treatment*						
Yes	ddm					
*Residence*						
Rural	Referent					
Urban	nsb	nsb	nsb	nsb	nsb	1.18 (0.89–1.56)
*Sex*						
Female	Referent					
Male	nsb	1.72 (1.08–2.72)	nsb	nsb	nsb	nsb
*Age*						
15–19	Referent					
20–24	1.59 (0.72–3.53)	nsb	0.93 (0.62–1.39)	1.37 (0.77–2.44)	1.44 (0.88–2.38)	1.47 (1.02–2.12)
25–34	2.79 (1.31–5.94)	nsb	1.52 (1.06–2.18)	2.19 (1.23–3.90)	2.01 (1.18–3.42)	1.48 (1.02–2.15)
35–49	3.12 (1.37–7.10)	nsb	1.70 (1.16–2.49)	1.35 (0.73–2.50)	1.97 (1.07–3.63)	1.74 (1.15–2.63)
50–64	2.03 (0.44–9.37)	nsb	1.84 (1.01–3.34)	3.99 (0.74–1.52)	1.74 (0.46–6.65)	2.56 (1.37–4.78)
*Zone*						
North West	Referent					
North Central	1.39 (0.68–2.85)	0.87 (0.45–1.70)	0.67 (0.47–0.95)	2.33 (1.04–5.20)	0.55 (0.34–0.88)	0.57 (0.41–0.79)
North East	2.72 (1.25–5.89)	0.89 (0.36–2.16)	0.54 (0.36–0.82)	1.01 (0.52–1.95)	1.27 (0.68–2.39)	0.78 (0.55–1.12)
South East	1.86 (0.79–4.37)	0.77 (0.34–1.71)	0.95 (0.6–1.49)	1.30 (0.47–3.62)	0.71 (0.39–1.27)	0.62 (0.39–0.98)
South South	0.63 (0.27–1.44)	1.99 (0.93–4.23)	1.39 (0.91–2.13)	0.87 (0.36–2.11)	1.81 (0.77–4.24)	0.89 (0.58–1.37)
South West	0.44 (0.20–0.95)	1.15 (0.58–2.28)	0.76 (0.46–1.24)	1.35 (0.69–2.65)	0.99 (0.55–1.79)	0.52 (0.32–0.84)
*Education*						
No formal education	Referent					
Primary/Qur'anic	2.41 (1.10–5.30)	1.39 (0.67–2.87)	1.08 (0.76–1.53)	1.34 (0.79–2.28)	1.66 (1.02–2.73)	1.48 (1.09–2.01)
Secondary	3.03 (1.31–6.98)	0.91 (0.44–1.89)	1.11 (0.77–1.59)	3.29 (1.74–6.20)	2.32 (1.37–3.94)	2.16 (1.56–2.99)
Higher	5.14 (2.03–13.0)	1.28 (0.55–2.99)	1.25 (0.8-1.95)	5.07 (1.98–13.0)	4.36 (2.07–9.18)	4.09 (2.55-6.58)
*Religion*						
Islam	Referent					
Other Christian	nsb	1.38 (0.74–2.57)	1.36 (1.01–1.85)	1.01 (0.53–1.87)	1.37 (0.86–2.17)	1.78 (1.34–2.35)
Catholics	nsb	1.70 (0.82–3.50)	1.33 (0.92–1.92)	1.16 (0.50–2.68)	1.62 (0.91–2.87)	1.87 (1.32–2.65)
Others	nsb	na	0.54 (0.18–1.67)	0.51 (0.13–1.98)	1.12 (0.18–6.97)	0.46 (0.18–1.15)
*Marital status*						
Currently married	Referent					
Widowed	nsb	nsb	nsb	nsb	0.76 (0.32–1.79)	0.87 (0.54–1.40)
Never married	nsb	nsb	nsb	nsb	1.44 (0.92–2.25)	0.99 (0.73–1.34)
*Economic status*						
Lowest	Referent					
Middle	na	na	1.17 (0.87–1.58)	na	na	1.76 (1.32–2.34)
Highest	na	na	1.15 (0.85–1.56)	na	na	2.07 (1.51–2.84)

^∗^Significant at 5%; ^a^Knowledge of STI; ^b^Knowledge of STI and HIV transmission and prevention and PMTCT; na, Not available; nsb, Not Significant at bivariate level; ddm, Dropped due to multicollinearity; aOR, adjusted Odds ratio; CI, Confidence Interval.

## Data Availability

The data is available on request.
